# Patient engagement during the transition from nondialysis‐dependent chronic kidney disease to dialysis: A meta‐ethnography

**DOI:** 10.1111/hex.13850

**Published:** 2023-08-28

**Authors:** Jinjie Liu, Yujun Zhou, Yanyao Tang, Jieling Chen, Jianying Li

**Affiliations:** ^1^ School of Nursing Sun Yat‐sen University Guangzhou China; ^2^ The First Affiliated Hospital Sun Yat‐sen University Guangzhou China

**Keywords:** chronic kidney disease, decision‐making, dialysis, life change event, meta‐ethnography, patient engagement

## Abstract

**Introduction:**

Patient engagement, encompassing both patient experience and opportunities for involvement in care, has been associated with increased patient satisfaction and the overall quality of care. Despite its importance, there is limited knowledge regarding patient engagement in the transition from nondialysis‐dependent chronic kidney disease (CKD) to dialysis‐dependent treatment. This systematic review employs meta‐ethnography to synthesize findings from qualitative studies examining patients' experiences of engagement during this transition, with the aim of developing a comprehensive theoretical understanding of patient engagement in the transition from nondialysis‐dependent CKD to dialysis.

**Methods:**

A systematic search of six databases, namely the Cochrane Library, PsycINFO, Scopus, Embase, PubMed and Web of Science was conducted to identify eligible articles published between 1990 and 2022. Meta‐ethnography was utilized to translate and synthesize the findings and develop a novel theoretical interpretation of ‘patient engagement’ during the transition to dialysis.

**Results:**

A total of 24 articles were deemed eligible for review, representing 21 studies. Patient engagement during a transition to dialysis was found to encompass three major domains: psychosocial adjustment, decision‐making and engagement in self‐care. These three domains could be experienced as an iterative and mutually reinforcing process, guiding patients toward achieving control and proficiency in their lives as they adapt to dialysis. Additionally, patient engagement could be facilitated by factors including patients' basic capability to engage, the provision of appropriate education, the establishment of supportive relationships and the alignment with values and resources.

**Conclusions:**

The findings of this review underscore the necessity of involving patients in transitional dialysis care, emphasizing the need to foster their engagement across multiple domains. Recommendations for future interventions include the provision of comprehensive support to enhance patient engagement during this critical transition phase. Additional research is warranted to explore the effects of various facilitators at different levels.

**Patient or Public Contribution:**

The studies included in our review involved 633 participants (547 patients, 14 family members, 63 healthcare providers and 9 managers). Based on their experiences, views and beliefs, we developed a deeper understanding of patient engagement and how to foster it in the future.

## 1 INTRODUCTION

The fifth stage of chronic kidney disease (CKD 5), also known as end‐stage kidney disease (ESKD), refers to low native kidney function, with an estimated glomerular filtration rate (eGFR) of less than 15 mL/min/1.73 m^2^.^1^ Patients with CKD 5 are required to receive kidney replacement therapy (KRT), which includes dialysis or kidney transplantation, or supportive care to sustain life. Dialysis is the most widely used KRT for patients with CKD 5 due to the limited availability of organ donations.^2^ Over 5.3 million people worldwide have CKD 5, and the global dialysis population is rapidly increasing.^3^, ^4^ The transition from nondialysis‐dependent CKD to dialysis therapy is a critical period in which a patient may experience profound physical, psychological and social challenges. The mortality risk is extremely high during the transition and reaches a peak in the first 2 months of the 24 months following the initiation of dialysis.^5^ Facilitating a smooth transition to dialysis is thus crucial to reducing mortality risks and improving psychosocial adjustment. Although suggestions on the timing of referral to transitional care vary, it is generally indicated that patients should receive transitional care and consider KRT planning if they are expected to undergo dialysis within the next 6–12 months, or their eGFR is below 30 mL/min/1.73 m^2^ (CKD 4 stage) with a progressive decline.^6^, ^7^ The patients' goals and preferences regarding treatment modality and access should be incorporated into the medical consultation.^8^, ^9^, ^10^ However, transitional care during the peridialysis period is inadequate and can result in an unplanned or emergency initiation of dialysis.^11^, ^12^

Patient engagement is defined as the desire and capability to actively participate in care in a way uniquely appropriate to the individual, in collaboration with healthcare providers, to maximize outcomes and improve the experiences of care.^13^ Patient engagement reflects the core value of person‐centred care, in which patients are encouraged to be actively involved in processing information, making and acting on decisions and managing self‐care.^14^, ^15^, ^16^ Promoting patient engagement has been recognized as crucial to improving patient experiences and outcomes.^9^ Understanding the experience of patient engagement during the transition to dialysis has significant implications.^9^, ^17^ When the disease progresses to CKD 5, most patients must make a range of complex decisions. These decisions include dialysis modality (i.e., haemodialysis or peritoneal dialysis) and specific choices concerning it such as the location of and access to the treatment. International guidelines recommend incorporating patients' goals and preferences regarding modality and access into the decision‐making process.^8^, ^9^, ^10^ However, evidence suggests that a large proportion of patients and their healthcare professionals do not work collaboratively to reach decisions regarding dialysis modality, and many patients feel unprepared to engage in the decision‐making process regarding dialysis.^18^, ^19^

While healthcare professionals often relate patient engagement to decision‐making, patient engagement includes a wider range of domains.^20^, ^21^ In addition to being involved in the decision‐making process, patients play a crucial role in making psychosocial adjustments and learning to manage their self‐care activities during the transition period. Patients often experience fear and ambivalence when they become aware of the irreversible nature of the disease.^22^ Supporting patients to cope with psychological and social distress and adjust to the profound changes in life is important while preparing for and initiating dialysis. Moreover, developing knowledge and abilities is vital to the management of self‐care for patients with CKD 5. For instance, patients who choose peritoneal dialysis should receive training to perform the dialysis until they are able to execute all exchange procedures by themselves. It is also important for patients to follow dietary and fluid restrictions, adhere to their medication regimens, and monitor symptoms and complications promptly, which are associated with better clinical outcomes and quality of life.^23^ Several factors contribute to patient engagement during the transition period, including patient‐related factors (e.g., education level, clinical status, motivation), health professional‐related factors (e.g., attitude toward patient engagement, communication), organization‐related factors (e.g., organizational climate and available resources), and lay community‐related factors (e.g., family and peer networks).^24^, ^25^

Previous reviews of patients with CKD 5 have mainly explored the experiences of patients who lived with maintenance dialysis,^26^, ^27^ but few reviews have specifically focused on the experiences of patients undergoing the transition from a nondialysis‐dependent state to a dialysis‐dependent state. Although some reviews have examined patients' experiences of engaging in decision‐making regarding dialysis initiation and modality selection, we argue that patient engagement during a transition period includes a wider range of domains in addition to decision‐making.^20^, ^21^ Patients play a crucial role in coping with the physical, psychological and social challenges and learning self‐care skills, which are important to adjust to profound new life.^28^, ^29^, ^30^ Moreover, these processes may interact. For instance, individuals with better psychosocial adjustment may be more likely to actively engage in decision‐making, whereas individuals who were maladaptive may be less likely to engage in decision‐making and improve self‐care skills. Given that patient engagement has been advocated as being pivotal to person‐centred care and thus quality of care, a deeper understanding is warranted concerning the experiences of patient engagement during the transition to dialysis. Thus, the objective of this study is to conduct a comprehensive literature search and use a meta‐ethnography to synthesize the qualitative studies that explored patients' experiences of engagement during the transition to dialysis to develop a theoretical understanding of patient engagement during the transition from nondialysis‐dependent CKD to dialysis. Such knowledge can assist in developing strategies that encourage patient engagement during this critical period to gain a better health outcome and quality of care.

## 2 METHODS

### 2.1 Study design

A meta‐ethnography was conducted to synthesize findings of experiences of patient engagement during the transition to dialysis. Meta‐ethnography, first described by Noblit and Hare,^31^ is an inductive and interpretative approach that differs from conventional narrative literature reviews or thematic synthesis, which primarily describe and aggregate findings. A meta‐ethnography compares and reinterprets the conceptual data (e.g., concepts, themes, metaphors) in a primary study and uses a translation synthesis method to create higher‐order or additional themes, which in turn guide the development of a deeper conceptual and theoretical understanding of a particular phenomenon.^32^ Meta‐ethnography has been widely used to generate evidence‐based insights for healthcare research and practice.^33^ In this review, we aim to synthesize the findings of qualitative studies exploring patients' experiences of engagement during the transition to dialysis to develop a theoretical understanding of patient engagement during this critical period. Thus, the meta‐ethnography is an appropriate method to synthesize findings and develop a theoretical understanding of the phenomenon we are interested in.

### 2.2 Search strategy and study selection

We systematically searched six databases (PubMed, Embase, PsycINFO, Web of Science, Scopus, and the Cochrane Library) to identify relevant articles published between January 1990 and September 2022. The keywords related to ‘patient engagement’ included patient participat*, patient involve*, patient empower*, patient activat*, patient engage*, life change event*, life experience*, life course*, disease manage*, self‐care, self‐management, decision mak*. The keywords related to “the transition to dialysis” included transition* care, transitional dialysis care, chronic kidney failure*, end‐stage kidney disease*, and end‐stage renal disease*, ESRD, ESKD, chronic renal failure*, dialysis, haemodialysis, peritoneal dialysis, renal replacement treatment, renal replacement therapy. The asterisk ‘*’ refers to a wildcard of letters. The detailed search strategy is presented in Supporting Information: Appendix S1. The references of the included articles were reviewed for additional articles.

After removing the duplicated records, we screened all identified records to determine their eligibility. Studies were included if they applied a qualitative study design to investigate experiences of patient engagement in patients with advanced or ESKD who were preparing for or undergoing the transition to dialysis. Also included were studies that examined experiences of patient engagement during the transition to dialysis from healthcare practitioners' and/or family members' perspectives. However, studies that focused only on the experiences during the long‐term maintenance dialysis were excluded as this review aimed to examine the experiences during the transition to dialysis. Studies that were not available in the English language were excluded. Titles and abstracts of all identified articles were screened by the main reviewer and a second independent reviewer (split between the other two reviewers) based on the eligibility criteria. Full texts were retrieved if the article passed the title/abstract screening or a disagreement arose on the relevance of a title/abstract screening. Two reviewers read the full texts independently and evaluated the eligibility of the articles. Any disagreements were resolved through discussions among the research team until a consensus was reached.

### 2.3 Data extraction

Data extraction was conducted by the first author using a customized spreadsheet. The following information was extracted to provide a context for the interpretation of each study: authors, year of publication, country of origin, characteristics of participants, sample sizes, research aim, research design, data collection methods and analytic methodology. We also extracted the participants' quotations and the primary authors' interpretations (e.g., concepts, themes) from each original study.

### 2.4 Appraisal of methodological quality

The Critical Appraisal Checklist for Qualitative Research developed by the Joanna Briggs Institute in 2017 was applied to assess the quality of the included articles.^34^ We used the 10‐item checklist to evaluate the methodological quality of the studies and to determine the extent to which each study had addressed the possibility of bias in its design, conduct and analysis. Two authors were responsible for conducting quality assessments independently. Disagreements were resolved through discussions until a consensus was reached.

### 2.5 Synthesis methodology

We applied the meta‐ethnography approach through a four‐step process to translate and synthesize the findings.^35^ First, the included articles were carefully read to provide an understanding of the participants' quotations (first‐order constructs) and the primary authors' interpretations (e.g., concepts, themes, metaphors). Concepts developed by the authors of the original studies, also known as second‐order constructs, were extracted from each original study and represented as the raw data for this meta‐ethnography synthesis. Second, concepts were compared and juxtaposed with each other to examine the relationships between concepts and to identify common recurring themes. Third, concepts were then translated from one study to another. The studies were arranged chronologically. Based on the concepts/themes from article one, the reviewer referred to the concepts/themes from article two, commenting on similarities between the first and second articles and how article two added to or diverged from article one. The synthesis of these two articles was then translated to article three, and the process was repeated until all articles were synthesized. Fourth, third‐order interpretations were generated by summarizing the translated second‐order constructs and considering the relationship between constructs. A line of argument, presented as a theoretical diagram, was developed to explain the relationship between the themes. This resulted in a higher level of interpretative synthesis and provided a comprehensive theoretical understanding of the experiences of patient engagement during the transition to dialysis.^32^

The first author led the meta‐ethnographic synthesis, and the research team met to review and discuss the synthesis process and develop the line of argument. The findings of this review have been reported in accordance with the reporting guidelines of Enhancing Transparency in Reporting the Synthesis of Qualitative Research Statement and the eMERGe reporting guidance to enhance the transparency of the findings.^35^, ^36^

## 3 RESULTS

### 3.1 Study selection

**Figure 1:**
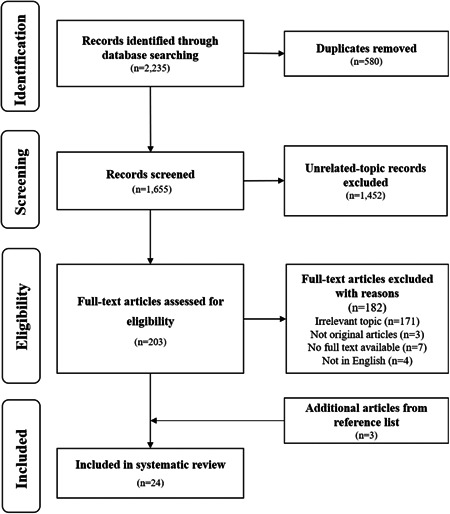
Flow diagram of study selection and exclusion.

Figure 1 summarizes the process of the study identification, selection and screening of the articles. The systematic review included 24 qualitative articles, representing 21 unique studies. The results of the quality assessment are presented in Supporting Information: Appendix S3. The quality of included articles was moderate‐to‐high overall, indicating acceptable reliability of the findings. However, only five studies illustrated the reflections on researchers' characters that might have influenced the research, and 11 studies stated the researchers' cultural or theoretical backgrounds.

### 3.2 Characteristics of the studies

A total of 633 participants in 24 articles were included in this review. Detailed information of the included articles (such as participant characteristics, study aims, study design and data collection methods) is provided in Supporting Information: Appendix S2. Of these articles, 17 described patients' values, beliefs and experiences; five explored perspectives from patients as well as caregivers and healthcare providers (nephrologists and nurses); and two employed only nephrologists and nurses as interviewees. The age of patients ranged from 20 to 90 years, while the length of work experience of the healthcare providers in nephrology ranged from 4 to 41 years. Most patients were at the fifth stage of CKD, and six studies also included patients with CKD 3b‐4 as participants. The duration of dialysis ranged from 0 to 12 months. These studies were conducted in a variety of countries as follows: Canada (*n* = 4), United States (*n* = 4), Australia (*n* = 3), New Zealand (*n* = 3), Denmark (*n* = 2), Sweden (*n* = 2), Norway (*n* = 1), Singapore (*n* = 1) and China (*n* = 1). The most frequent type of qualitative design was phenomenology (*n* = 5), followed by explorative design (*n* = 4), grounded theory (*n* = 3), exploratory descriptive design (*n* = 2), generic qualitative design (*n* = 2) and ethnography (*n* = 1). Seven articles did not provide such information about the type of qualitative design.

### 3.3 Derived themes

Table 1 presents the third‐order constructs (themes and subthemes), translated second‐order constructs, and article sources. Four themes emerged from the included studies: psychosocial adjustment, experiences of decision‐making, engagement in self‐care and facilitators for patient engagement. We presented example quotations in Supporting Information: Appendix S4.

**Table 1:** Derived themes of meta‐ethnography.

Themes (third‐order constructs)	Subthemes (third‐order constructs)	Translated second‐order constructs	Sources (article no.)
Psychosocial adjustment	Emotional distress	Shock	5, 6, 9, 13, 17, 19
		Regret	9, 14
		Fear	4, 5, 6, 10, 14, 23
		Shame and suicidal thoughts	5, 6, 14
	Cognitive processing	Deny	5, 13, 14
		Self‐blame or blame others	9, 14
		Realistic expectation and acceptance	4, 9, 12, 13, 24
		Positive refocusing	4, 5, 24
		Positive reappraisal	4, 9, 15, 24
		Refocus on planning	1, 9, 24
		Catastrophizing	6, 9
	Social function	Employment	5, 23
		Domestic life	3, 6, 13, 23
		Social activities	5, 6, 9, 13, 23
Experiences of decision‐making	Struggling to make a decision	Hesitated and confused	16, 19
		Delay in making decisions	7, 10, 12, 16
		An urgent choice	10, 11
	Shared model	Patient autonomy	2, 4, 7, 11, 17
		Building mutual trust	7, 8, 14, 17
		Iterative decision process	19
		Necessary information and guidance	1, 4, 7, 18, 19
		Decision aid	19
	Paternalistic model	Patient lacks decisional power	8, 11, 21
		Inappropriate information	8, 11, 22
		Depending on professional expertize	2, 3, 11, 12
	Informed model	Decision‐making power in patients	21
		Thorough information	21
		Physicians' influence	11, 21
	Aspects of modality choice	Illness experiences	1, 8, 14, 15
		Impacts on life	1, 3, 8, 12, 15
		Concerns regarding treatment	1, 8, 15
		Resources	8, 15
Engagement in self‐care	Modifying health behaviours	Adhering to diet and fluid recommendation	5, 13, 18, 21, 23
		Adhering to medication regimen	18, 21
	Monitoring complications	Measuring basic signs	23
		Staying alert for symptoms	5, 6, 18, 23
	Keeping regular dialysis	Taking part in planning care and treatment	18
		Self‐mastering dialysis operation	18, 20, 21
Facilitators for patient engagement	Basic capability to engage	Patient's physical function	20
		Cognitive function	2, 18, 20, 24
		Psychological function	3, 13, 14, 23
	Appropriate education	More personalized information	2, 3, 10, 17, 18
		Timely and common language	2, 3, 4, 20
	Supportive relationships	Therapeutic alliance	2, 4, 10, 18, 19, 20
		Social support	1, 4, 5, 11, 14, 17, 19
		Peer support	1, 4, 5, 7, 8, 14, 19, 20, 22
	Values and resources	Healthcare team's consensus of ‘patient engagement’	20, 21, 22
		Time for patient engagement	17, 20, 21, 22
		Programme for patient engagement	10, 17, 20, 21

#### 3.3.1 Psychosocial adjustment

Psychosocial adjustment describes the process by which an individual responds to the demands of an illness to move toward a state of person‐environment congruence.^37^ Psychosocial adjustment is characterized by emotional, cognitive and social adjustment. Patients with CKD 5 usually experience emotional distress. Many of these patients did not realize that the symptoms and signs indicated a deterioration of kidney function until they met the doctors and received the diagnosis.^38^, ^39^ Regardless of whether the patients were aware of the disease before its progression, patients were usually shocked to discover that they would need a life‐long therapy.^40^ Some patients expressed regret for not having proactively taken preventive measures at earlier stages.^41^ Many patients mentioned feelings of fear, including a fear of the unknown, a fear of being a burden to family members, and a fear of death.^42^ Perceiving oneself as a burden to the family could result in shame and even suicidal thoughts.^39^, ^41^, ^43^ For example, a patient reported that ‘It was the fear of the unknown. I thought I was going to be an invalid for the rest of my life and… I was thinking about leaving my husband and leaving my son’.^43^

Patients used cognitive adjustment strategies to regulate feelings and make sense of their experiences. Seven cognitive processing styles were identified from the included articles: denial, self‐blame or blaming others, realistic expectation and acceptance, positive refocusing, catastrophizing, positive reappraisal and refocusing on planning.^44^ Many patients were initially in denial of the diagnosis.^40^ They may subconsciously delay the dialysis until the disease had developed into an acute state and only then started emergency dialysis. For example, the patient said ‘Initially, I was just rejecting it. I did not want to go for dialysis. I did not want to fix my fistula. I will try all medication and see whether it helps’.^39^ Patients blamed themselves or others, such as doctors, for failing to monitor their kidney function.^41^ However, the continuous deterioration in their physical condition forced the patients to go from denial to accepting the necessity of dialysis. Some patients reported that their doctors would tell them, ‘If you don't go on dialysis, you die’.^45^ Although patients found the idea of endless dialysis and the threat of death painful, it seems that accepting dialysis would be a realistic way to prevent the condition from getting even worse. Over the initial period of dialysis, patients often described a sense of isolation and deprivation. They fixated on the negative impacts of dialysis and had various catastrophizing thoughts, such as ‘I can't get out of it anymore’, or ‘I lose the day that I have the dialysis’, which indicates maladaptation.^38^, ^43^ Positive refocusing refers to thinking about pleasant and positive aspects rather than the original event.^44^ Patients at younger ages or with fewer economic concerns were more likely to look forward to a kidney transplant, whether it may or may not be forthcoming.^39^, ^46^, ^47^ Some patients reappraised the situation in a more positive way and attempted to make sense of their experiences.^38^, ^46^, ^47^, ^48^ Keeping regular dialysis has made the patients perceive a sense of purpose in daily life. Some reported that dialysis reminded them of the limitations of life, which made them value the time outside dialysis and engage in activities that could create more value. For example, a patient explained, ‘I really try to fill my time when I'm at home, it's really precious time. I work out what I'm going to do and who I'm going to ring’.^38^ Refocusing on planning is also cited as a useful coping strategy to alleviate feelings of uncertainty about their future. Patients may think about how to handle the circumstance and develop new daily routines instead of engaging in avoidance. They forced themselves ‘stop worrying about the little things and worry about something that really matters’.^47^ Making attempts in terms of diet, schedule and travel adjustment increased the patients' confidence and sense of control in adapting to a life with dialysis.^38^, ^49^

During the transition to dialysis, patients experience social demands regarding employment, family roles and social activities. Patients who were employed before dialysis were at risk of losing their jobs since dialysis was time‐consuming.^39^ Patients revealed that dialysis had a profound impact on the entire family. Due to the constraints of the disease and treatment, patients may contribute less to and demand more from the family.^40^, ^43^, ^50^ Patients often found it hard to adjust to the new family roles and feel ambivalent about receiving help from others. An old widow dependent on her busy daughter explained ‘You've lost your independence… you depend on other people all the time’.^43^ Some patients felt that their close relationships could not withstand the challenges of the illness and its associated consequences.^51^ Additionally, dialysis reduced the time and energy available for the patients to participate in social activities as they normally would, which may result in less social support and a sense of loneliness.^38^ Some patients also distanced themselves from others when they began dialysis.^43^ The patient explained: ‘I didn't want to talk to nobody. I didn't want to associate with the family in any way. I just wanted to be left alone’. However, managing social demands is essential to mastering a new life with maintenance dialysis.

#### 3.3.2 Experience in decision‐making

Most patients with CKD 5 need to prepare for KRT and make a range of treatment‐related decisions, such as the modality of dialysis, time to start dialysis and procedures for preparing for dialysis. Three subthemes were identified regarding the experience of decision‐making during the transition to dialysis from the included studies: struggling to make a decision, the decision‐making model (a paternalistic model, an informed model or a shared model), and considerations in choosing a treatment.

The decisional conflict was highly prevalent in patients who were newly diagnosed with CKD 5.^52^, ^53^ Patients may delay making decisions for various reasons. Some felt overwhelmed by having to consider the choices, whereas others found it hard to make a decision since no solution was optimal.^45^, ^52^, ^54^ However, after repeatedly being informed of the necessity of dialysis, patients eventually realized that they had to accept that dialysis might be the only option for sustaining life. As one patient described, ‘If I don't begin dialysis, I might as well plan my funeral’.^55^ Nevertheless, some patients put off making a decision to start dialysis until other members of the healthcare team had to make the decision during an emergency.^42^

The three main types of decision‐making models are the paternalistic, the informed, and the shared models. The models are described in terms of information exchange, discussion and deliberation of treatment preferences, and the decision on the treatment to be implemented.^56^ The paternalistic model is a traditional decision‐making model. During the transition to dialysis, some patients perceived that they lacked power in decision‐making and felt pressure when they attempted to ask questions or articulate their concerns and preferences.^57^ Previous illness experiences may cause some patients to merely listen passively and wait for a clinical directive.^45^, ^50^, ^55^, ^58^ Moreover, some patients found it difficult to understand the complex information which contained so much medical jargon, whereas other patients reported that they received only limited information or had brief discussions with the healthcare professionals, which increased the likelihood of depending on clinicians to make a decision based on their expertize.^45^, ^55^, ^57^ These patients attempted to engage in the decision‐making with limited success, such as ‘I did not want to [start dialysis]. I was quarrelling with my physician, because he was really… interested in having my dialysis. I didn't like it… They didn't give me very much information’.^55^ In contrast to the paternalistic model, patient engagement plays a central role in decision‐making from the informed approach. Healthcare professionals provided tailored information and evidence on the benefits and risks of the treatment options, which enabled patients to make informed decisions.^59^ Patients are fully responsible for deliberating on the information and making a final decision on their own. A range of clinical guidelines calls for the use of the shared decision‐making model in making a decision on dialysis initiation and choice of modality.^60^ The shared decision‐making model values patients' autonomy and their willingness to engage in decision‐making. The patient would realize that ‘I made the decision. I didn't want to give that to the boys, [they] have a lot on their mind and I have to make the decision myself. It's up to me. I had to say it’.^55^ The model emphasizes that both healthcare professionals and patients should be involved and develop mutual trust to work collaboratively during the decision‐making process. The decision process was iterative, wherein both parties exchanged the necessary information (e.g., treatment information, resources and patients' preferences) and deliberated over the choices until an agreement was reached.^53^, ^61^ Decision aids, such as videos and value clarification tools were useful in facilitating shared decision‐making.^53^

A key decision during the transition to dialysis is modality choice, which includes the type of dialysis (i.e., haemodialysis or peritoneal dialysis) and the location of dialysis (i.e., home or centre). For decisions on modality, the included articles indicated that patients typically considered the trade‐offs of each modality with regard to four aspects: illness experiences, impacts on life, treatment‐related concerns and resources. When considering modality options, patients reflected on their past experiences of being in the hospital, receiving treatment and dealing with other comorbidities.^41^ These past experiences of illness may shape their beliefs and views of disease and thus influence their choices concerning dialysis. Patients also considered treatment suggestions from those who had similar experiences (e.g., peers or relatives).^41^, ^48^, ^49^, ^57^ A major concern regarding modality choice is the impact of dialysis on personal life (i.e., quality of sleep, dietary habits, employment, the possibility for travel, and family life (i.e., economic, physical and psychological burdens). Patients preferred convenient treatment that would enable them to lead a normal life that entailed a minimum of life disruptions to themselves and their families. Some patients chose home dialysis, which allowed flexibility in planning the dialysis schedule.^49^ For example, nocturnal dialysis at home enabled the patients that ‘when he gets up in the morning, he'll start his day as per normal so that's not going to affect his daily routine’.^48^ Patients who required a high level of medical support were more likely to choose facility‐based haemodialysis to reduce the burden on their families.^50^ In terms of social interaction, some patients thought that performing home‐based dialysis themselves would allow them more freedom to maintain social engagement.^57^ On the other hand, patients who chose haemodialysis might develop a bond with clinical staff and other patients, which contributed to a sense of belonging.^48^ Patients may have concerns about treatment when considering the modality choice. For example, they worried about whether they had adequate physical capability or manual dexterity to perform dialysis well on their own, or whether it was safe to perform the dialysis independently.^49^, ^57^ Patients also considered the available resources that could support a realistic choice, such as whether the home environment was suitable for a particular type of dialysis, whether transportation to the hospital was convenient, and whether they could afford the costs of a particular type of dialysis.^57^

#### 3.3.3 Engagement in self‐care

A third main theme identified from the included studies is engagement in self‐care. Given the changing health condition, it is vital that patients with CKD 5 gain the ability to manage self‐care activities while undergoing the transition to dialysis. The three subthemes identified were modifying health behaviours, monitoring complications and keeping regular dialysis. Dialysis imposes restrictions on diet and fluid intake, and nonadherence may increase the risks of fluid overload, hyperkalaemia and hyperphosphatemia. Patients need to make modifications to their daily dietary behaviours to adhere to fluid and dietary restrictions called for during the transition. Some well‐adapted patients followed special homemade special diets in adherence to recommendations from professionals.^51^ Some could adjust their diets in accordance with their indicators, for instance, a patient reported ‘I thought to myself that I could eat more if I didn't increase my weight; I could eat less if my blood potassium was not controlled’.^51^ Patients also need to comply with the prescribed medication regimens, which entails a minimum level of patient engagement.^62^ Another task is to monitor the physical symptoms and laboratory data associated with the effectiveness of treatment and disease progression, such as oedema, fatigue, thirst and muscle cramps.^39^, ^43^, ^51^, ^62^ Patients' monitoring behaviours (measuring weight and blood pressure, checking the function of the vascular fistula, etc.) with timely response to abnormal indications contributed to preventing complications.^51^ From the initiation of dialysis, patients must keep regular dialysis. Patients were encouraged to take part in the planning and practice of their care and treatment, including planning and complying with the dialysis schedule, calculating the amount of fluids that would be extracted during each dialysis session, and the self‐manipulation of dialysis machine at home.^59^, ^62^, ^63^

#### 3.3.4 Facilitators for patient engagement

More than half of the included articles explored the factors that facilitate patient engagement during the transition to dialysis. Four factors were identified based on the synthesis of the evidence, including basic capability, appropriate education, supportive relationships, values and resources. The basic capability to engage describes the physical, cognitive and psychological functions that enable individuals to engage in their treatment. Health literacy is a central indicator of the cognitive capability associated with patient engagement, which refers to the ability of individuals to obtain, process, understand and act on health information and services to make appropriate health decisions.^64^ Patients with low health literacy found it hard to understand their situation despite having received a great deal of medical information. This could reduce their engagement in sharing preferences or goals with the healthcare team.^62^, ^64^ Patients who are physically capable and independently functional are more likely to perform basic and instrumental activities of daily living and learn other self‐care activities to manage their disease. Some patients, especially those in the acute phase, had severe symptoms (e.g., fatigue, oedema or shortness of breath), which restricted them from being able to make certain medical decisions or engaging in self‐care activities.^64^ In addition, patients built inner strength and empowerment through the support and comfort they gained through their families, beliefs, faith or cultural identity.^40^, ^41^, ^51^ Inner strength and self‐confidence encouraged the patient to make psychosocial adjustment during the transition, which also fostered their engagement in seeking information, making decisions and changing behaviours to manage their health condition.^47^, ^50^, ^58^, ^62^

Appropriate patient education is another important factor that affects patient engagement. Patients mentioned that more comprehensive and concrete information about the disease or treatment (e.g., prognostic information, possible treatment options, the dialysis process) could alleviate fear and a sense of uncertainty and foster involvement in decision‐making and prepare them for the future.^50^, ^58^ The information should be balanced in terms of presenting both benefits and risks of each modality, which allows patients to make choices that are aligned with their goals and preferences, rather than providing biased information that favours the healthcare professionals' preferences (e.g., a physician specialized in haemodialysis may feel uncertain about dealing with peritoneal dialysis). The educational programme should be personalized and delivered at the right time to foster patient engagement.^50^, ^58^, ^65^ The process of information provision should take into account the ability of patients to process the information as well as their needs and preferences. Patients commonly found it hard to process professional information while still in shock about or in denial of their diagnosis.^46^ Giving patients some time to understand the information could enhance their engagement in decision‐making. One healthcare provider stated, ‘You can talk more about it the next time and let it take time because patients are different and can be affected in various ways’.^62^ A shared language between healthcare professionals and patients also fostered the willingness of patients to exchange information and communicate with their healthcare providers, whereas complex medical jargon created misunderstandings and confusion.^63^

Patients value the role of supportive relationships with the healthcare team, family members and other patients with CKD 5 in enhancing their engagement. The therapeutic alliance between healthcare providers and patients could be facilitated by maintaining openness and continuity in communication, respecting patients' values and preferences, being sensitive towards the patients' emotional stress, being empathetic and having mutual understanding.^46^, ^55^, ^62^, ^63^ For example, it was important that the healthcare providers listened to and addressed patients' questions to build a therapeutic alliance.^53^ Patients might regain control and self‐worth through assistance and affirmation from the healthcare team, which empowered them to share feelings, participate in shared decision‐making and take action.^58^ Patients acknowledged the instrumental and emotional support from family and friends. Patients received information and discussed it with their family and friends to increase their understanding of their conditions and make treatment decisions.^53^, ^55^ Family members provided tangible support, such as reminding the patient to take their medication regularly and assisting in performing dialysis, which can be of great help during the early stages of care.^39^, ^46^ Emotional support from family and friends could help relieve patients' distress and facilitate their ability to cope with and adjust to the transition. Additionally, patients highlighted the benefit of knowing other patients with CKD 5. Most patients wanted to seek information and learn from other patients with CKD 5 because they could ask ‘what they actually want to ask’.^66^ Interacting with other patients facing similar challenges enabled them to shift perspectives and it alleviated the sense of isolation. Patients also felt reassured by comparing their situations with those of their peers. Some patients perceived that their situation was not the worst, which could instill optimism regarding their ability to cope with future treatment. Moreover, some patients became sympathetic toward those who were worse off.^46^

At the organizational level, values and available resources were identified to influence patient engagement during the transition to dialysis. Primarily, a lack of consensus on ‘patient engagement’ in a healthcare team can hinder the involvement of a patient in implementing transitional dialysis care. If the healthcare team did not believe that patient engagement would add value to the implementation of their care, they were less likely to support patients to improve their health literacy, provide thorough information, take the time and effort to build therapeutic alliances or develop education and peer programmes to facilitate patient engagement.^59^, ^63^ A lack of resources is also a major obstacle to promoting patient engagement. Many physicians and nurses experienced time constraints that inhibited them from taking the time to talk with patients and foster mutual understanding.^42^, ^63^, ^65^ Moreover, the availability of structural programmes for promoting patient engagement varied between different organizations. Some organizations offered education programmes and/or peer programmes to increase patients' capacities, provided information and emotional support, and guided patients to engage in decision‐making and self‐care.^42^, ^61^ However, such programmes were not available in some organizations.

## 4 PROPOSED MODEL

**Figure 2:**
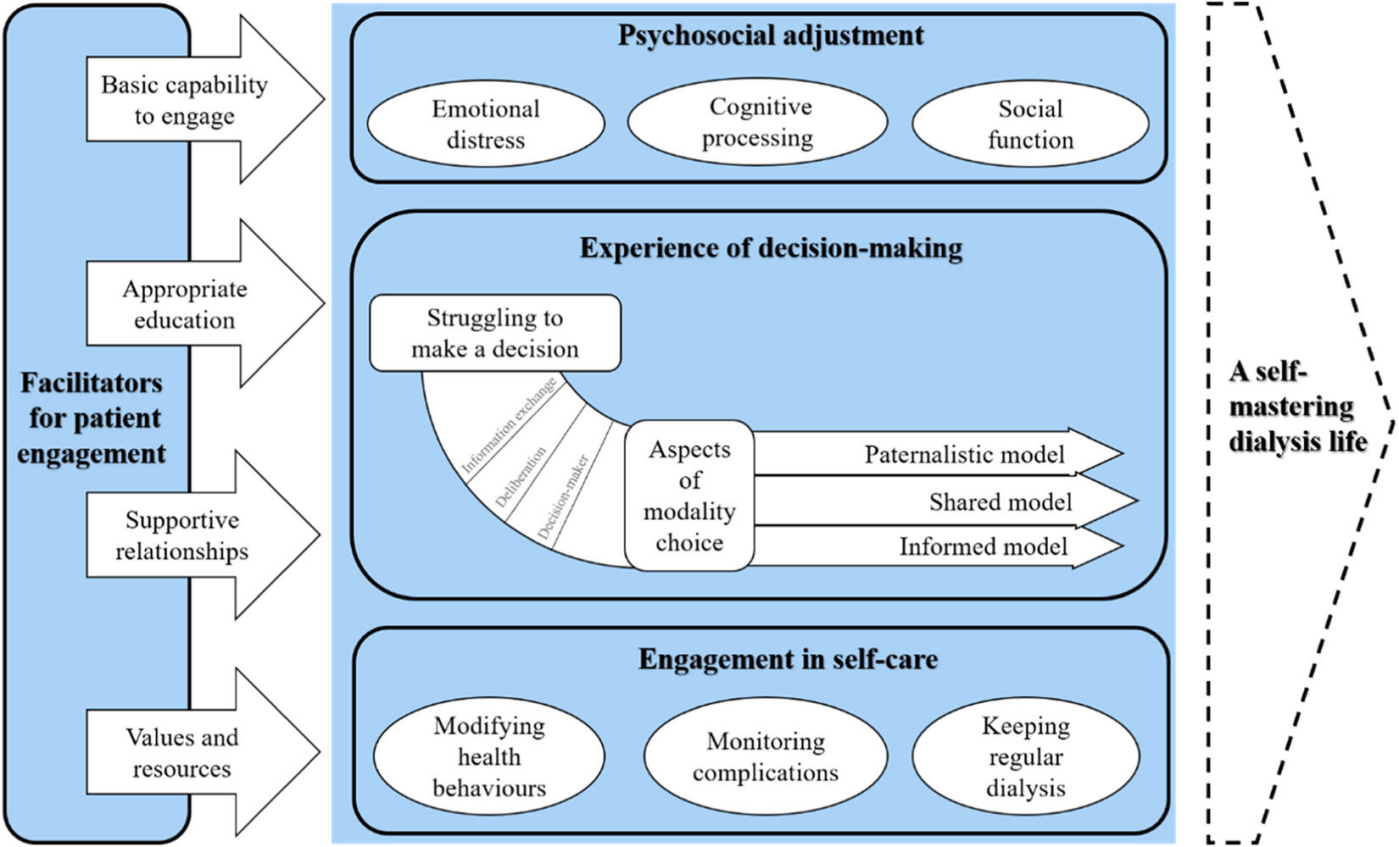
The model of patient engagement during the transition to dialysis.

By synthesizing the findings from the 24 qualitative articles, we developed a model that describes the experiences of patient engagement during the transition to dialysis and illustrates the factors associated with patient engagement (Figure 2). Patients experience a wide range of physical, psychological and social challenges and need to engage in three major domains during this transition. These comprise making psychological and social adjustment to the changes, engaging in decision‐making, and engaging in managing self‐care activities. The three domains could be experienced as an iterative and mutually reinforcing process. For instance, the improved psychosocial adjustment may enable the patients to engage in decision‐making actively, whereas engaging in the process of shared decision‐making may also provide support for patients and help them rebuild a sense of control and hope for the future. The synthesis also indicates that patient engagement in transitional care is affected by four major factors: basic capability to engage, appropriate education, supportive relationships, values and resources. These factors could be grouped into three levels. At the individual level, patients' physical, cognitive and psychological capacities are associated with their ability to engage in their care. At the social level, healthcare providers may develop supportive relationships with patients and deliver targeted education to them, which is crucial to facilitating patient engagement. Additionally, supportive relationships with families, friends and peer patients also foster patient engagement. At the organizational level, values and available resources influence the promotion of patient engagement in practical care. Patients are encouraged to engage in different domains during the transition to dialysis, contributing to achieving control and proficiency in their lives as they adapt to dialysis.

## 5 DISCUSSION

To our best knowledge, this represents the first study to conduct a comprehensive literature search and develop a theoretical understanding of patient engagement during the transition from nondialysis‐dependent CKD to dialysis therapy based on existing evidence. Patient engagement during the transition to dialysis included three major domains: psychosocial adjustment, decision‐making and self‐care. Moreover, the facilitation of patient engagement may be influenced by four factors at the individual, social and organizational levels, including patients' basic capability to engage, targeted education, the establishment of supportive relationships and alignment with values and resources.

The current review identified psychosocial adjustment as one of the central themes during the transition to dialysis, which has also been identified as an important theme in previous reviews on the experiences of patients living with maintenance dialysis.^26^, ^27^ However, the emphasis on psychosocial adjustment in the present context pertains to the acceptance of the necessity for dialysis. It entails a process where patients may transition from a state of ‘rejection’ to ‘acceptance’ and from ‘disengagement’ to ‘engagement’. Engagement in decision‐making is another central theme identified in the included studies. The findings of this review share similarities with previous reviews on decision‐making during the transition to dialysis.^21^, ^67^, ^68^, ^69^, ^70^ While diverse models of decision‐making exist (e.g., the paternalistic model, the informed model and the shared model), this review, consistent with previous reviews, underscores the significance of recognizing patients' needs, beliefs and preferences and enabling their active participation in decision‐making through tailored education, effective communication and equitable relationships. Additionally, the theme of self‐care was also highlighted. Compared to reviews of patients living with maintenance dialysis that emphasized maintaining long‐term self‐care to adhere to treatment requirements,^71^, ^72^ this review indicates that patients made efforts to acquire knowledge and develop a range of self‐care skills during the transition period. It is noteworthy that these three processes may be interrelated, and those patients who successfully engage in them are likely to attain a state of self‐mastery in life with dialysis.

Our review provides novel insights into facilitating patient engagement at the individual, social and organizational levels. The factors at these three levels can interact with each other. Patients possessing stronger physical, cognitive and psychological capacities are more likely to adopt a proactive approach to their medical responsibilities, seeking adequate knowledge and available resources to enhance their autonomy.^73^ Additional support at the social and organizational levels might further strengthen patients' abilities to engage.^74^, ^75^, ^76^

Twenty‐four articles were identified and included in this review. The study sample encompassed a wide variation in age and geographic area and incorporated perspectives from patients, family members and healthcare providers, thus enhancing the representativeness of the findings. The stages of CKD varied across the studies, with some focusing solely on CKD stage 5, while others examined both stages 5 and 3b–4. There is no consensus regarding the optimal timing to engage patients in preparation for the transition to dialysis.^6^, ^7^ Patients' goals, preferences and motivations to engage in healthcare may vary across different stages of kidney disease. For instance, patients with CKD stage 4 may have more time and energy to explore various dialysis modalities with their healthcare team compared to those with CKD stage 5. However, patients nearing dialysis within the next 6–12 months may exhibit a stronger motivation to engage in medical decision‐making. Further research is recommended to investigate engagement in transitional care across different stages of CKD.

This study has several limitations. First, it solely includes qualitative studies, focusing on in‐depth insights into people's perspectives, feelings and beliefs and excludes quantitative studies that might contribute to a more comprehensive theoretical understanding of patient engagement. Second, it should be noted that the method of meta‐ethnography itself has certain shortcomings.^77^ The inclusion of qualitative studies conducted in diverse contexts might lead to an oversimplification of complex issues by reducing consideration of specific differences. Nevertheless, the establishment of a team and regular meetings to discuss the data analysis process may enhance the rigour and reliability of the results. Lastly, the exclusion of grey literature and articles not reported in English may limit insights into patient engagement.

Existing literature in this field has predominantly concentrated on involving patients in the decision‐making process during the transition to dialysis.^78^ Several predialysis patient education programmes and dialysis decision aids have been developed for patients with advanced and/or ESKD to facilitate their engagement in decision‐making.^79^, ^80^, ^81^, ^82^, ^83^ However, our synthesized model reveals that engagement in peridialysis care encompasses multiple domains in addition to decision‐making. Therefore, recognizing patients' comprehensive needs and devising integrated intervention strategies is essential to enabling engagement in various domains, including psychosocial adjustment, participation in decision‐making and the development of self‐care skills. Additionally, patient engagement may be enhanced by concerted efforts across individual, social and organizational levels. For instance, individuals may focus on enhancing their physical, cognitive and psychological functions, while families or caregivers can provide companionship and comfort to reinforce supportive relationships. Furthermore, healthcare administrators or clinical managers could concentrate on optimizing resource allocation and the education of clinical professionals to facilitate patient engagement. Future quantitative studies could explore the relationships between different components of patient engagement and their associations with factors at multiple levels.

## 6 CONCLUSIONS

The findings of this review indicate that patients often encounter profound physical, psychological and social challenges during the transition to dialysis. A comprehensive model was formulated to depict the experiences of patient engagement and its correlated factors. During this transition, patients play a crucial role in engaging in three principal domains: psychosocial adjustment, decision‐making and developing self‐care skills. The model further emphasizes that patient engagement is associated with four factors (basic capability to engage, appropriate education, supportive relationships and alignment with values and resources) at three different levels. It is recommended that future clinical practice incorporate the voices of patients, families, healthcare caregivers and organization managers in developing and designing interventions to foster patient engagement. Further quantitative research is recommended to examine the relationships between the three domains of patient engagement and evaluate the impact of multilevel factors on patient engagement during the transition to dialysis.

## AUTHOR CONTRIBUTIONS

Jinjie Liu, Jianying Li and Jieling Chen conducted the study conceptualization. Jinjie Liu conducted, Yujun Zhou and Yanyao Tang conducted article search, screening, data extraction and quality appraisal. Jinjie Liu conducted data analysis and all authors were involved in discussion of the analysis. Jinjie Liu drafted the first version of the manuscript. Jinjie Liu, Jieling Chen and Jianying Li revised the manuscript. All authors reviewed and approved the final manuscript.

## CONFLICT OF INTEREST STATEMENT

The authors declare no conflict of interest.

## Supporting information

Supporting information.

Supporting information.

Supporting information.

Supporting information.

## Data Availability

Data sharing is not applicable to this article as no datasets were generated or analysed during the current study.

## References

[1] Lv JC , Zhang LX . Prevalence and disease burden of chronic kidney disease. Adv Exp Med Biol. 2019;1165:3‐15. 10.1007/978-981-13-8871-2_1 31399958

[2] Thurlow JS , Joshi M , Yan G , et al. Global epidemiology of end‐stage kidney disease and disparities in kidney replacement therapy. Am J Nephrol. 2021;52(2):98‐107. 10.1159/000514550 33752206 PMC8057343

[3] Bello AK LA , Lunney M , Osman MA , et al. Global Kidney Health Atlas: a Report by the International Society of Nephrology on the Global Burden of End‐Stage Kidney Disease and Capacity for Kidney Replacement Therapy and Conservative Care across World Countries and Regions. International Society of Nephrology; 2019.

[4] Hill NR , Fatoba ST , Oke JL , et al. Global prevalence of chronic kidney disease ‐ a systematic review and meta‐analysis. PLoS ONE. 2016;11(7):e0158765. 10.1371/journal.pone.0158765 PMC4934905 27383068

[5] Foley RN , Chen SC , Solid CA , Gilbertson DT , Collins AJ . Early mortality in patients starting dialysis appears to go unregistered. Kidney Int. 2014;86(2):392‐398. 24522495 10.1038/ki.2014.15

[6] Lok CE , Huber TS , Lee T , et al. KDOQI clinical practice guideline for vascular access: 2019 update. Am J Kidney Dis. 2020;75(suppl 2):S1‐S164. 10.1053/j.ajkd.2019.12.001 32778223

[7] National Kidney Foundation . KDOQI clinical practice guideline for hemodialysis adequacy: 2015 update. Am J Kidney Dis. 2015;66(5):884‐930. 10.1053/j.ajkd.2015.07.015 26498416

[8] Bowman B , Zheng S , Yang A , et al. Improving incident ESRD care via a transitional care unit. Am J Kidney Dis. 2018;72(2):278‐283. 10.1053/j.ajkd.2018.01.035 29510919

[9] Chan CT , Blankestijn PJ , Dember LM , et al. Dialysis initiation, modality choice, access, and prescription: conclusions from a kidney disease: improving global outcomes (KDIGO) controversies conference. Kidney Int. 2019;96(1):37‐47. 10.1016/j.kint.2019.01.017 30987837

[10] Vandecasteele SJ , Kurella Tamura M . A patient‐centered vision of care for ESRD: dialysis as a bridging treatment or as a final destination? J Am Soc Nephrol. 2014;25(8):1647‐1651. 10.1681/asn.2013101082 24833125 PMC4116069

[11] Atieh AS , Shamasneh AO , Hamadah A , Gharaibeh KA . Predialysis nephrology care amongst Palestinian hemodialysis patients and its impact on initial vascular access type. Ren Fail. 2020;42(1):200‐206. 10.1080/0886022x.2020.1727512 32506996 PMC7048207

[12] Milkowski A , Prystacki T , Marcinkowski W , et al. Lack or insufficient predialysis nephrology care worsens the outcomes in dialyzed patients—call for action. Ren Fail. 2022;44(1):946‐957. 10.1080/0886022x.2022.2081178 35652160 PMC9176675

[13] Higgins T , Larson E , Schnall R . Unraveling the meaning of patient engagement: a concept analysis. Patient Educ Couns. 2017;100(1):30‐36. 10.1016/j.pec.2016.09.002 27665500

[14] Jamal N . Reflections on patient‐centered care: from the perspective of a young otolaryngologist. Otolaryngol Head Neck Surg. 2017;157(4):543‐544. 10.1177/0194599817721455 28719758

[15] Murali NS , Deao CE . Patient engagement. Prim Care. 2019;46(4):539‐547. 10.1016/j.pop.2019.07.007 31655750

[16] Tang C , Lorenzi N , Harle CA , Zhou X , Chen Y . Interactive systems for patient‐centered care to enhance patient engagement. J Am Med Inform Assoc. 2016;23(1):2‐4. 10.1093/jamia/ocv198 26912537 PMC7814929

[17] Berger JR , Jaikaransingh V , Hedayati SS . End‐stage kidney disease in the elderly: approach to dialysis initiation, choosing modality, and predicting outcomes. Adv Chronic Kidney Dis. 2016;23(1):36‐43. 10.1053/j.ackd.2015.08.005 26709061

[18] Bombard Y , Baker GR , Orlando E , et al. Engaging patients to improve quality of care: a systematic review. Implement Sci. 2018;13(1):98. 10.1186/s13012-018-0784-z 30045735 PMC6060529

[19] Martinsson C , Uhlin F , Wenemark M , Eldh AC . Preference‐based patient participation for most, if not all: a cross‐sectional study of patient participation amongst persons with end‐stage kidney disease. Health Expect. 2021;24(5):1833‐1841. 10.1111/hex.13323 34337836 PMC8483194

[20] Shi Y , Li W , Duan F , et al. Factors promoting shared decision‐making in renal replacement therapy for patients with end‐stage kidney disease: systematic review and qualitative meta‐synthesis. Int Urol Nephrol. 2022;54(3):553‐574. 10.1007/s11255-021-02913-8 34159522 PMC8831292

[21] Yu, X , Nakayama, M , Wu, MS , et al. Shared decision‐making for a dialysis modality. Kidney Int Rep, 2022;7(1):15‐27. 10.1016/j.ekir.2021.10.019 35005310 PMC8720663

[22] Niu H , Liu J . The psychological trajectory from diagnosis to approaching end of life in patients undergoing hemodialysis in China: a qualitative study. Int J Nurs Sci. 2017;4(1):29‐33. 10.1016/j.ijnss.2016.10.006 31406714 PMC6626075

[23] Van Eck Van Der Sluijs A , Vonk S , Van Jaarsveld BC , Bonenkamp AA , Abrahams AC . Good practices for dialysis education, treatment, and ehealth: a scoping review. PLoS One. 2021;16(8):e0255734. 10.1371/journal.pone.0255734 34379654 PMC8357118

[24] Chia JMX , Goh ZS , Seow PS , et al. Psychosocial factors, intentions to pursue arteriovenous dialysis access and access outcomes: a cohort study. Am J Kidney Dis. 2021;77(6):931‐940. 10.1053/j.ajkd.2020.09.019 33279557

[25] Havas K , Douglas C , Bonner A . Person‐centred care in chronic kidney disease: a cross‐sectional study of patients' desires for self‐management support. BMC Nephrol. 2017;18(1):17. 10.1186/s12882-016-0416-2 28086812 PMC5237219

[26] Bayhakki U , Hatthakit U . Lived experiences of patients on hemodialysis: a meta‐synthesis. Nephrol Nurs J. 2012;39(4):295‐304. 23061114

[27] Polaschek N . The experience of living on dialysis: a literature review. Nephrol Nurs J. 2003;30(3):303‐309. 12861898

[28] Evans M , Lopau K . The transition clinic in chronic kidney disease care. Nephrol Dial Transplant. 2020;35(suppl 2):ii4‐ii10. 32162667 10.1093/ndt/gfaa022 PMC7066544

[29] Kalantar‐Zadeh K , Kovesdy CP , Streja E , et al. Transition of care from pre‐dialysis prelude to renal replacement therapy: the blueprints of emerging research in advanced chronic kidney disease. Nephrol Dial Transplant. 2017;32(suppl 2):ii91‐ii98. 28201698 10.1093/ndt/gfw357 PMC5837675

[30] Molnar MZ , Streja E , Sumida K , et al. Pre‐ESRD depression and post‐ESRD mortality in patients with advanced CKD transitioning to dialysis. Clin J Am Soc Nephrol. 2017;12(9):1428‐1437. 28679562 10.2215/CJN.00570117 PMC5586564

[31] Noblit GW , Hare RD . Meta‐Ethnography: Synthesizing Qualitative Studies. Sage Publications Inc.; 1988.

[32] Cahill M , Robinson K , Pettigrew J , Galvin R , Stanley M . Qualitative synthesis: a guide to conducting a meta‐ethnography. Br J Occup Ther. 2018;81(3):129‐137. 10.1177/0308022617745016

[33] Sattar R , Lawton R , Panagioti M , Johnson J . Meta‐ethnography in healthcare research: a guide to using a meta‐ethnographic approach for literature synthesis. BMC Health Serv Res. 2021;21(1):50. 10.1186/s12913-020-06049-w 33419430 PMC7796630

[34] Lockwood C , Munn Z , Porritt K . Qualitative research synthesis: methodological guidance for systematic reviewers utilizing meta‐aggregation. Int J Evid Based Healthc. 2015;13(3):179‐187. 10.1097/XEB.0000000000000062 26262565

[35] France EF , Cunningham M , Ring N , et al. Improving reporting of meta‐ethnography: the eMERGe reporting guidance. J Adv Nurs. 2019;75(5):1126‐1139. 10.1111/jan.13809 30644123 PMC7594209

[36] Tong A , Flemming K , McInnes E , Oliver S , Craig J . Enhancing Transparency in Reporting the Synthesis of Qualitative Research: ENTREQ. BMC Med Res Methodol. 2012;12:181. 10.1186/1471-2288-12-181 23185978 PMC3552766

[37] Derogatis LR . The psychosocial adjustment to illness scale (PAIS). J Psychosom Res. 1986;30(1):77‐91. 10.1016/0022-3999(86)90069-3 3701670

[38] Gullick J , Monaro S , Stewart G . Compartmentalising time and space: a phenomenological interpretation of the temporal experience of commencing haemodialysis. J Clin Nurs. 2017;26(21‐22):3382‐3395. 10.1111/jocn.13697 28001331

[39] Lai AY , Loh APP , Mooppil N , Krishnan DSP , Griva K . Starting on haemodialysis: a qualitative study to explore the experience and needs of incident patients. Psychol Health Med. 2012;17(6):674‐684. 10.1080/13548506.2012.658819 22397505

[40] Nagpal N , Boutin‐Foster C , Melendez J , et al. Experiences of patients undergoing dialysis who are from ethnic and racial minorities. J Ren Care. 2017;43(1):29‐36. 10.1111/jorc.12185 27977065 PMC6152882

[41] Walker RC , Walker S , Morton RL , Tong A , Howard K , Palmer SC . Māori patients' experiences and perspectives of chronic kidney disease: a New Zealand qualitative interview study. BMJ Open. 2017;7(1):e013829. 10.1136/bmjopen-2016-013829 PMC5253593 28104711

[42] Henry SL , Munoz‐Plaza C , Garcia Delgadillo J , Mihara NK , Rutkowski MP . Patient perspectives on the optimal start of renal replacement therapy. J Ren Care. 2017;43(3):143‐155. 10.1111/jorc.12202 28393467

[43] Monaro S , Stewart G , Gullick J . A ‘lost life’: coming to terms with haemodialysis. J Clin Nurs. 2014;23(21‐22):3262‐3273. 10.1111/jocn.12577 24810661

[44] Garnefski N , Kraaij V , Spinhoven P . Negative life events, cognitive emotion regulation and emotional problems. Pers Individ Differ. 2001;30(8):1311‐1327. 10.1016/S0191-8869(00)00113-6

[45] Lovell S , Walker RJ , Schollum JB , Marshall MR , McNoe BM , Derrett S . To dialyse or delay: a qualitative study of older New Zealanders' perceptions and experiences of decision‐making, with stage 5 chronic kidney disease. BMJ Open. 2017;7:014781. 10.1136/bmjopen-2016-014781 PMC5372046 28360253

[46] Mitchell A , Farrand P , James H , Luke R , Purtell R , Wyatt K . Patients' experience of transition onto haemodialysis: a qualitative study. J Ren Care. 2009;35(2):99‐107. 10.1111/j.1755-6686.2009.00094.x 19432855

[47] Thorsteinsdottir B , Espinoza Suarez NR , Curtis S , et al. Older patients with advanced chronic kidney disease and their perspectives on prognostic information: a qualitative study. J Gen Intern Med. 2022;37:1031‐1037. 10.1007/s11606-021-07176-8 35083651 PMC8971255

[48] Wong B , Venturato L , Oliver MJ , Quinn RR , Ravani P , Holroyd‐Leduc J . Selection of peritoneal dialysis among older eligible patients with end‐stage renal disease. Nephrol Dial Transplant. 2016;32(2):gfw367. 10.1093/ndt/gfw367 28186576

[49] Tweed AE , Ceaser K . Renal replacement therapy choices for pre‐dialysis renal patients. Br J Nurs. 2005;14(12):659‐664. 10.12968/bjon.2005.14.12.18287 16010217

[50] Davison SN , Simpson C . Hope and advance care planning in patients with end stage renal disease: qualitative interview study. BMJ. 2006;333(7574):886. 10.1136/bmj.38965.626250.55 16990294 PMC1626305

[51] Szu LY , Tsao LI , Chen SC , Ho ML . Self‐participation experiences among well‐adapted hemodialysis patients. Healthcare. 2021;9(12):1742. 10.3390/healthcare9121742 34946468 PMC8701990

[52] Campbell‐Crofts S , Stewart G . How perceived feelings of “wellness” influence the decision‐making of people with predialysis chronic kidney disease. J Clin Nurs. 2018;27(7‐8):1561‐1571. 10.1111/jocn.14220 29240277

[53] Finderup J , Dam Jensen J , Lomborg K . Evaluation of a shared decision‐making intervention for dialysis choice at four Danish hospitals: a qualitative study of patient perspective. BMJ Open. 2019;9(10):e029090. 10.1136/bmjopen-2019-029090 PMC6803133 31630101

[54] Erlang AS , Nielsen IH , Hansen HOB , Finderup J . Patients experiences of involvement in choice of dialysis mode. J Ren Care. 2015;41(4):260‐267. 10.1111/jorc.12141 26417666

[55] Ladin K , Lin N , Hahn E , Zhang G , Koch‐Weser S , Weiner DE . Engagement in decision‐making and patient satisfaction: a qualitative study of older patients' perceptions of dialysis initiation and modality decisions. Nephrol Dial Transplant. 2016;32(8):gfw307. 10.1093/ndt/gfw307 PMC5837335 27576590

[56] Charles C , Whelan T , Gafni A . What do we mean by partnership in making decisions about treatment? BMJ. 1999;319(7212):780‐782. 10.1136/bmj.319.7212.780 10488014 PMC1116606

[57] Walker RC , Howard K , Morton RL , Palmer SC , Marshall MR , Tong A . Patient and caregiver values, beliefs and experiences when considering home dialysis as a treatment option: a semi‐structured interview study. Nephrol Dial Transplant. 2016;31(1):133‐141. 10.1093/ndt/gfv330 26346314

[58] Davison SN . Facilitating advance care planning for patients with end‐stage renal disease: the patient perspective. Clin J Am Soc Nephrol. 2006;1(5):1023‐1028. 10.2215/cjn.01050306 17699322

[59] Andersen‐Hollekim T , Landstad BJ , Solbjør M , Kvangarsnes M , Hole T . Nephrologists' experiences with patient participation when long‐term dialysis is required. BMC Nephrol. 2021;22(1):58. 10.1186/s12882-021-02261-w 33593314 PMC7885613

[60] Galla JH . Clinical practice guideline on shared decision‐making in the appropriate initiation of and withdrawal from dialysis. J Am Soc Nephrol. 2000;11(7):1340‐1342. 10.1681/asn.V1171340 10864592

[61] Cassidy BP , Getchell LE , Harwood L , Hemmett J , Moist LM . Barriers to education and shared decision making in the chronic kidney disease population: a narrative review. Can J Kidney Health Dis. 2018;5:205435811880332. 10.1177/2054358118803322 PMC6236635 30542621

[62] Årestedt L , Martinsson C , Hjelm C , Uhlin F , Eldh AC . Patient participation in dialysis care—a qualitative study of patients' and health professionals' perspectives. Health Expect. 2019;22(6):1285‐1293. 10.1111/hex.12966 31560830 PMC6882253

[63] Årestedt L , Martinsson C , Hjelm C , Uhlin F , Eldh AC . Context factors facilitating and hindering patient participation in dialysis care: a focus group study with patients and staff. Worldviews Evid Based Nurs. 2020;17(6):457‐464. 10.1111/wvn.12452 32696513

[64] Speros C . Health literacy: concept analysis. J Adv Nurs. 2005;50(6):633‐640. 10.1111/j.1365-2648.2005.03448.x 15926968

[65] Cassidy BP , Harwood L , Getchell LE , Smith M , Sibbald SL , Moist LM . Educational support around dialysis modality decision making in patients with chronic kidney disease: qualitative study. Can J Kidney Health Dis. 2018;5:205435811880332. 10.1177/2054358118803323 PMC6178119 30327720

[66] Stoye A , Zimmer JM , Girndt M , Mau W . The role of different nephrology experts in informed shared decision‐making for renal replacement therapy. J Ren Care. 2021;48:177‐184. 10.1111/jorc.12397 34482634

[67] Hussain JA , Flemming K , Murtagh FEM , Johnson MJ . Patient and health care professional decision‐making to commence and withdraw from renal dialysis: a systematic review of qualitative research. Clin J Am Soc Nephrol. 2015;10(7):1201‐1215. 10.2215/cjn.11091114 25943310 PMC4491298

[68] Kim EY , Son YJ . Developing a conceptual model of older patients' decision‐making process in choosing dialysis or conservative care using meta‐ethnography. J Adv Nurs. 2022;78(1):1‐13. 10.1111/jan.14945 34227152

[69] Raj R , Thiruvengadam S , Ahuja KDK , Frandsen M , Jose M . Discussions during shared decision‐making in older adults with advanced renal disease: a scoping review. BMJ Open. 2019;9(11):e031427. 10.1136/bmjopen-2019-031427 PMC6887047 31767590

[70] Rosansky SJ , Schell J , Shega J , et al. Treatment decisions for older adults with advanced chronic kidney disease. BMC Nephrol. 2017;18(1):200. 10.1186/s12882-017-0617-3 28629462 PMC5477347

[71] Costa Pessoa NR , de Souza Soares Lima LH , Dos Santos GA , de Queiroz Frazão CMF , Sousa CN , Ramos VP . Self‐care actions for the maintenance of the arteriovenous fistula: an integrative review. Int J Nurs Sci. 2020;7(3):369‐377. 10.1016/j.ijnss.2020.06.007 32817861 PMC7424158

[72] Lambert K , Mullan J , Mansfield K . An integrative review of the methodology and findings regarding dietary adherence in end stage kidney disease. BMC Nephrol. 2017;18(1):318. 10.1186/s12882-017-0734-z 29061163 PMC5653982

[73] Sedin A , Isaksson J , Patel H . The experience of transitioning into life‐sustaining treatment: a systematic literature review. J Ren Care. 2022;49:158‐169. 10.1111/jorc.12439 35932286

[74] Sauvé C , Vandyk AD , Bourbonnais FF . Exploring the facilitators and barriers to home dialysis: a scoping review. Nephrol Nurs J. 2016;43(4):295‐308. 30550056

[75] Ahn JW , Lee SM , Seo YH . Factors associated with self‐care behavior in patients with pre‐dialysis or dialysis‐dependent chronic kidney disease. PLoS One. 2022;17(10):e0274454. 10.1371/journal.pone.0274454 36227926 PMC9560058

[76] Neumann D , Lamprecht J , Robinski M , Mau W , Girndt M . Social relationships and their impact on health‐related outcomes in peritoneal versus haemodialysis patients: a prospective cohort study. Nephrol Dial Transplant. 2018;33(7):1235‐1244. 10.1093/ndt/gfx361 29370430

[77] Campbell R , Pound P , Morgan M , et al. Evaluating meta‐ethnography: systematic analysis and synthesis of qualitative research. Health Technol Assess. 2011;15(43):1‐164. 10.3310/hta15430 22176717

[78] Harwood L , Clark AM . Understanding pre‐dialysis modality decision‐making: a meta‐synthesis of qualitative studies. Int J Nurs Stud. 2013;50(1):109‐120. 10.1016/j.ijnurstu.2012.04.003 22560169

[79] Devoe DJ , Wong B , James MT , et al. Patient education and peritoneal dialysis modality selection: a systematic review and meta‐analysis. Am J Kidney Dis. 2016;68(3):422‐433. 10.1053/j.ajkd.2016.02.053 27125246

[80] Green JA , Ephraim PL , Hill‐Briggs FF , et al. Putting patients at the center of kidney care transitions: PREPARE NOW, a cluster randomized controlled trial. Contemp Clin Trials. 2018;73:98‐110. 10.1016/j.cct.2018.09.004 30218818 PMC6679594

[81] Harwood L , Clark AM . Dialysis modality decision‐making for older adults with chronic kidney disease. J Clin Nurs. 2014;23(23‐24):3378‐3390. 10.1111/jocn.12582 24646195

[82] Koch‐Weser S , Porteny T , Rifkin DE , et al. Patient education for kidney failure treatment: a mixed‐methods study. Am J Kidney Dis. 2021;78(5):690‐699. 10.1053/j.ajkd.2021.02.334 33894282

[83] Subramanian L , Zhao J , Zee J , et al. Use of a decision aid for patients considering peritoneal dialysis and in‐center hemodialysis: a randomized controlled trial. Am J Kidney Dis. 2019;74(3):351‐360. 10.1053/j.ajkd.2019.01.030 30954312

