# Supplementary material for: Patient engagement during the transition from nondialysis‐dependent chronic kidney disease to dialysis: A meta‐ethnography
Source: Health Expect. 2023 Aug 28;26(6):2191–204. doi: 10.1111/hex.13850 (PMC10632643; doi:10.1111/hex.13850)
Supplement: Supplementary file 1 — Supporting information. [file HEX-26--s004.docx]

**Appendix 1 Search strategy**

| Boolean algebra | MeSH Term | Entry terms |
| --- | --- | --- |
| OR | Patient Participation | Patient Participation  Patient Involvement  Patient Empowerment  Patient Participation Rates  Patient Participation Rate  Patient Activation  Patient Engagement |
|  | Stakeholder Participation | Stakeholder Participation  Stakeholder Engagement  Stakeholder Role |
|  | Life change events | Life change events  Life Change Event  Life Experiences  Life Experience  Life Course  Life Courses  Lifespan |
|  | Disease management | Disease managements |
|  | Self care | Self-management |
|  | Decision Making, Shared | Shared Decision Making |
| AND |  |  |
| OR | Transition care | Pre-dialysis care  Pre-dialysis education  Predialysis care  Predialysis education  Transitional dialysis care  Transition clinic |
|  | Kidney Failure, Chronic | End-Stage Kidney Disease  End Stage Kidney Disease  Chronic Kidney Failure  End-Stage Renal Disease  End Stage Renal Disease  End-Stage Renal Failure  Chronic Renal Failure  ESRD  ESKD |
|  | Renal Replacement Therapy | Dialysis  Renal dialysis  Hemodialysis  Peritoneal dialysis  Renal replacement treatment  Renal replacement therapy |
