# Supplementary material for: Patient engagement during the transition from nondialysis‐dependent chronic kidney disease to dialysis: A meta‐ethnography
Source: Health Expect. 2023 Aug 28;26(6):2191–204. doi: 10.1111/hex.13850 (PMC10632643; doi:10.1111/hex.13850)
Supplement: Supplementary file 2 — Supporting information. [file HEX-26--s001.docx]

**Appendix 2 Characteristics of the included articles**

| **No** | **Author (Year)** | **Country** | **CKD characteristics** | **Sample size (mean age)** | **Data Collection Method** | **Research Design** | **Data Analysis Method** | **Aim** |
| --- | --- | --- | --- | --- | --- | --- | --- | --- |
| 1 | Tweed & Ceaser (2005) | UK | CKD 5 (likely to require renal replacement therapy within a time frame of 2–18 months) | 9 (54) | semi-structured interviews | phenomenology | interpretative phenomenological analysis (IPA) | to explore the decision-making processes of pre-dialysis patients to elucidate how these choices were made. |
| 2 | Davison (2006) | Canada | ①CKD 5 (were clinically expected to require dialysis within the next 12 months); ②HD patients; ③PD patients | 24 (64) | in-depth interviews | ethnography | constant comparative and iterative analyses | to determine the perspectives of patients with ESRD of the salient elements of ACP discussions. |
| 3 | Davison & Simpson (2006) | Canada | CKD 5 (were clinically expected to require dialysis within the next 12 months) | 19 (65) | in-depth interviews | generic qualitative  design | inductive analysis | to understand hope in the context of advance care planning from the perspective of patients with end stage renal disease. |
| 4 | Mitchell et al. (2009) | UK | CKD 5 (had started haemodialysis within the previous six months.) | 10 (-) | Semi-structured interviews | - | Interpretive content analysis | to identify factors identified by patients as helpful in the transition onto haemodialysis |
| 5 | Lai, Loh, Mooppil, Krishnan, & Griva (2012) | Singapore | incident haemodialysis patients (defined as CKD 5 patients undergoing haemodialysis for a period of six months or less) | 13 (52) | semi-structured interviews | - | interpretative phenomenological analysis | to identify the main concerns and needs encountered by incident patients via the exploration of their lived experiences during the early months on haemodialysis. |
| 6 | (Monaro, Stewart, & Gullick, 2014) | Australia | ①patients (within three months of HD commencement); ②caregivers | ①11 (62.5);  ②5(64) | semi-structured interviews | Heideggerian phenomenology | interpretative phenomenological analysis | to describe the essence of the lived experience of patients and families in the early phase of long-term haemodialysis therapy |
| 7 | (Erlang, Nielsen, Hansen, & Finderup, 2015) | Denmark | CKD 4-5 (have chosen a dialysis mode, but not yet started the treatment) | 9 (-) | semi- structured individual interviews | a qualitative method including both phenomenology and hermeneutics | systematic text condensation | to gather information about how patients experienced involvement in the decision-making process of RRT just after they have made the decision, and before starting dialysis. |
| 8 | (Walker et al., 2016) | New Zealand | ①CKD 4-5 (had received formal education about dialysis treatment options or had commenced dialysis within the previous 12 months); ②caregivers (an informal caregiver for a family member or close friend on dialysis.) | ①43;  ②9;  (55) | semi-structured interviews | grounded theory | thematic analysis | to describe patient and caregiver values, beliefs and experiences when considering home dialysis, to inform strategies to align policy and practice with patients’ needs |
| 9 | (Gullick, Monaro, & Stewart, 2017) | Australia | ①CKD 5 (had commenced dialysis within the previous three months); ②close family members | ①11 (62.5);  ②5(64) | Semi- structured interviews | Heidegger's hermeneutic phenomenology | interpretative phenomenological analysis | to interpret the spatio-temporal experience of people with end-stage kidney disease and their families in the first months of haemodialysis. |
| 10 | (Henry, Munoz-Plaza, Garcia Delgadillo, Mihara, & Rutkowski, 2017) | America | CKD 5 (initiated HD between January 2008 and September 2008) | 168 (65) | semi-structured phone interviews | - | deductive and inductive methodological approaches | to characterise the experiences of patients beginning RRT. |
| 11 | (Ladin et al., 2017) | America | CKD 5 (receiving maintenance outpatient dialysis >1 month) | 31 (76) | semi-structured interviews. | - | codebook | to examine patient perspectives of the dialysis initiation process (i.e., decision to start dialysis) and the relationship between patient engagement and treatment satisfaction, which is inadequately studied in the US context |
| 12 | (Lovell et al., 2017) | New Zealand | CKD 5 (had undergone dialysis education but had not started dialysis or recently started dialysis within the past 6 months) | 17 (75) | semi -structured interviews | - | framework analysis | to examine the experiences of older adults (aged ≥65 years) living with chronic kidney disease (CKD) as they chose whether or not to begin dialysis or continue with conservative management. |
| 13 | (Nagpal et al., 2017) | America | CKD 5 (who had been on dialysis for more than six months) | 36 (53) | Open-ended interviews | descriptive phenomenology | A series of standard qualitative techniques | to understand the psychosocial context of living with ESKD among black and Latino adults who reside in a medically underserved community. |
| 14 | (Walker et al., 2017) | New Zealand | CKD 4–5D (were either nearing the need for dialysis or had started dialysis within the previous 12 months.) | 13 (59) | semi-structured interviews | adapted grounded theory | thematic analysis | to explore and describe Māori (the indigenous people of New Zealand) patients’ experiences and perspectives of chronic kidney disease (CKD), as these are largely unknown for indigenous groups with CKD. |
| 15 | (Wong et al., 2017) | Canada | pre-dialysis patients and those who had initiated dialysis within the preceding 6 months | 16 (-) | semi-structured interviews | - | line-by-line textual analysis | to determine the patient-perceived factors that influence ESRD patients' choice of dialysis modality among older ESRD patients who are deemed eligible for both PD and HD |
| 16 | (Campbell-Crofts & Stewart, 2018) | Australia | CKD3b-5 (not yet commenced any form of RRT) | 12 (-) | intermittent semi-structured interviews | an exploratory qualitative descriptive design | thematic analysis | to identify the subjective meanings attached to decisions made by people living with chronic kidney disease as they consider their transition to renal replacement therapy. |
| 17 | (Cassidy et al., 2018) | Canada | CKD 5 (on either IC-HD, PD, or Home-HD who started dialysis within 6 months of the study) | 12 (-) | in-depth, semi -structured interviews | generic qualitative  design | content analysis | to understand the dialysis modality decision-making process through exploration of the pre-dialysis patient experience to better inform the educational process. |
| 18 | (Årestedt, Martinsson, Hjelm, Uhlin, & Eldh, 2019) | Sweden | ①patients (have encountered at least five dialysis sessions); ②staff (have performed dialysis at least for six months); ③managers (be in charge of first‐line issues, including measures for quality of care at unit level.) | ①15(-);  ②18(-);  ③9(-) | focus group discussions | explorative study | content analysis | to conceptualize patient participation in dialysis care, depicting patients' and healthcare professionals' perspectives |
| 19 | (Finderup, Dam Jensen, & Lomborg, 2019) | Denmark | eGFR＜20mL/min measured by a 24-hour urine test | 29 (-) | Semi- structured individual interviews | descriptive and explorative method | systematic text condensation | to gain a clear insight into how the patients experienced the impact of SDM-DC on their involvement in the decision-making process. |
| 20 | (Årestedt, Martinsson, Hjelm, Uhlin, & Eldh, 2020) | Sweden | ①patients (having had at least five dialysis treatments); ②healthcare providers (employed for more than 6 months in dialysis care); ③managers of the dialysis unit. | ①15(-);  ②18(-);  ③9(-) | focus group discussions | an explorative qualitative design | content analysis | to identify what patients and HCPs depict in terms of their perceptions of enablers and barriers for patient participation in dialysis care. |
| 21 | (Andersen-Hollekim, Landstad, Solbjør, Kvangarsnes, & Hole, 2021) | Norway | nephrologists or physicians (with a minimum of one-year experience in dialysis care.) | 10(-) | semi-structured interview | explorative study | interpretative phenomenological analysis | to explore nephrologists' experiences with patient participation in different phases of the end-stage renal disease trajectory for working-age individuals who require dialysis. |
| 22 | (Stoye, Zimmer, Girndt, & Mau, 2021) | Germany | nephrologists and nurses (from different dialysis units all over Germany) | 35 (-) | problem‐centred interviews | explorative study | structuring and summary content analysis | to explore nephrologists' and nurses' perspectives regarding their involvement in shared decision‐making when choosing renal replacement therapy |
| 23 | (Szu, Tsao, Chen, & Ho, 2021) | China (Taiwan) | well-adaptive haemodialysis patients (had been undergoing haemodialysis three times a week for at least one year) | 32 (61.72) | Open-ended interview | grounded theory | comparative methods | to describe haemodialysis patients’ perspectives on integrating haemodialysis into a new life regarding self-participation experience. |
| 24 | (Thorsteinsdottir et al., 2022) | America | CKD 3b-5 (not yet commenced any form of RRT) | 28 (77.7) | Semi-structured telephone or in-person interviews | - | inductive content analysis. | to understand the prognostic information preferences expressed by older patients with CKD. |
| Abbreviations and definitions: CKD 5, chronic kidney disease stage 5 patients; CKD 4-5, chronic kidney disease stage 4-5 patients. | | | | | | | | |
| -: the information was not mentioned in the article. | | | | | | | | |
