# Supplementary material for: Patient engagement during the transition from nondialysis‐dependent chronic kidney disease to dialysis: A meta‐ethnography
Source: Health Expect. 2023 Aug 28;26(6):2191–204. doi: 10.1111/hex.13850 (PMC10632643; doi:10.1111/hex.13850)
Supplement: Supplementary file 3 — Supporting information. [file HEX-26--s003.docx]

Appendix 3 Quality appraisal of the included articles

| **Article no.** | **1** | **2** | **3** | **4** | **5** | **6** | **7** | **8** | **9** | **10** | **11** | **12** | **13** | **14** | **15** | **16** | **17** | **18** | **19** | **20** | **21** | **22** | **23** | **24** |
| --- | --- | --- | --- | --- | --- | --- | --- | --- | --- | --- | --- | --- | --- | --- | --- | --- | --- | --- | --- | --- | --- | --- | --- | --- |
| 1. Is there congruity between the stated philosophical perspective and the research methodology? | √ | √ | - | √ | - | √ | √ | - | √ | - | - | - | √ | √ | - | √ | √ | √ | √ | √ | √ | - | √ | - |
| 2. Is there congruity between the research methodology and the research question or objectives? | √ | √ | √ | √ | √ | √ | √ | √ | √ | √ | √ | √ | √ | √ | √ | √ | √ | √ | √ | √ | √ | √ | √ | √ |
| 3. Is there congruity between the research methodology and the methods used to collect data? | √ | √ | √ | √ | √ | √ | √ | √ | √ | × | √ | √ | √ | √ | √ | √ | √ | √ | √ | √ | √ | √ | √ | √ |
| 4. Is there congruity between the research methodology and the representation and analysis of data? | √ | √ | √ | √ | √ | √ | √ | √ | √ | √ | √ | √ | √ | √ | √ | √ | √ | √ | √ | √ | √ | √ | √ | √ |
| 5. Is there congruity between the research methodology and the interpretation of results? | √ | √ | √ | √ | √ | √ | √ | √ | √ | √ | √ | √ | √ | √ | √ | √ | √ | √ | √ | √ | √ | √ | √ | √ |
| 6. Is there a statement locating the researcher culturally or theoretically? | × | × | × | × | × | √ | √ | × | √ | √ | × | × | × | √ | √ | × | √ | × | × | √ | √ | × | √ | √ |
| 7. Is the influence of the researcher on the research, and vice- versa, addressed? | × | × | × | × | × | √ | √ | √ | √ | √ | × | × | × | √ | √ | × | × | × | × | × | √ | × | × | √ |
| 8. Are participants, and their voices, adequately represented? | √ | √ | √ | √ | √ | √ | √ | √ | √ | √ | √ | √ | √ | √ | √ | √ | √ | √ | √ | √ | √ | √ | √ | √ |
| 9. Is the research ethical according to current criteria or, for recent studies, and is there evidence of ethical approval by an appropriate body? | √ | √ | √ | √ | √ | √ | √ | √ | √ | √ | √ | √ | √ | √ | √ | √ | √ | √ | √ | √ | √ | √ | √ | √ |
| 10. Do the conclusions drawn in the research report flow from the analysis, or interpretation, of the data? | √ | √ | √ | √ | √ | √ | √ | √ | √ | √ | √ | √ | √ | √ | √ | √ | √ | √ | √ | √ | √ | √ | √ | √ |
| Result | B | B | B | B | B | A | A | B | A | B | B | B | B | A | B | B | B | B | B | B | A | B | B | B |

“-“ means “unclear”
