# Supplementary material for: Patient engagement during the transition from nondialysis‐dependent chronic kidney disease to dialysis: A meta‐ethnography
Source: Health Expect. 2023 Aug 28;26(6):2191–204. doi: 10.1111/hex.13850 (PMC10632643; doi:10.1111/hex.13850)
Supplement: Supplementary file 4 — Supporting information. [file HEX-26--s002.docx]

**Appendix 4** Development of third-order constructs in the meta-ethnographic synthesis (with example quotations)

| **Themes**  **(Third-order constructs)** | **Sub-themes**  **(Third-order constructs)** | **Translated Second-order constructs** | **Sources (article no.)** | **Example quotations** |
| --- | --- | --- | --- | --- |
| **Psychosocial adjustment** | **Emotional distress** | Shock | 5，6，9，13，17，19 | *5: “I feel that I cannot breathe, so I went for a check-up at the hospital and water had already accumulated in my lungs . . . I was shocked. I have a kidney problem.”*  *13: “I was in denial. I said ‘no that’s not me, that’s for other people’. Then a year later, my feet started to swell. I couldn’t walk 10 feet. I could not put on shoes or tie them up. I had excessive weight gain. Eventually, when they did it, they did emergency dialysis.”* |
|  |  | Regret | 9，14 | *9: “It was probably a little bit of me... I wasn’t ready to do it and Dr X. went along with that: he felt that I wasn’t ready to actually take that extra step... To do it before it gets too bad.”* |
|  |  | Fear | 4，5，6，10，14，23 | *5: “Initially when I was diagnosed, I felt that my life is so short. Within those two weeks I felt depressed . . . I felt that I would just die anytime.”*  *10: “How was I going to cope with everyday life? How I was going to cope with work, and how was I going to cope with my self-esteem realizing that I wasn’t in control of my own body.”* |
|  |  | Shame and suicidal thoughts | 5，6，14 | *6: “It was the fear of the unknown. I thought I was going to be an invalid for the rest of my life and... I was thinking about leaving my husband and leaving my son.”* |
|  | **Cognitive processing** | Deny | 5，13，14 | *5: “Initially, I was just rejecting it. I did not want to go for dialysis. I did not want to fix my fistula. I will try all medication and see whether it helps.”* |
|  |  | Self-blame or blame others | 9，14 | *14: “I hated going to the doctor, being told off, but now when I think back, I was dumb, I didn’t go, didn’t take my insulin, didn’t take my pills, drunk too much, smoked, you know everything you shouldn’t do.”* |
|  |  | Realistic expectation and acceptance | 4，9，12，13，24 | *4: “Your life doesn’t go on...I’m well aware of my life expectancy but it’s things you want to do and it’s a fact...All the regrets, you put things into perspective.”*  *12:* *“If you don’t go on dialysis, you die.”* |
|  |  | Positive refocusing | 4，5，24 | *5: “I hope to maintain my current health state while waiting for a kidney. It’s the best to get a kidney transplant.”* |
|  |  | Positive reappraisal | 4，9，15，24 | *9: “I really try to fill my time when I’m at home, it’s really precious time. I work out what I’m going to do and who I’m going to ring.”*  *15:* *“That means I have a purpose every day. I mean, 3 times a week that I have an appointment to go.”* |
|  |  | Refocus on planning | 1，9，24 | *9: “For Janice, it was a case of determining what was possible day-to-day within the scope of her changed function: ‘I’ve got to get into a routine and work out what I can and can’t do’.”*  *24: “**I would probably stop worrying about the little things and worry about somethin’ that really matters.”* |
|  |  | Catastrophizing | *6，9* | *9: “You lose the day that you have the dialysis and it seems to involve both of us. Her [wife’s] time is taken up as well as my time.”* |
|  | **Social function** | Employment | 5，23 | *5: “Now, I am able to perform carpentry and sell my own creations. My wife no longer has to endure many hardships... I feel more like a man now that I have more self-worth and a sense of self-accomplishment.”* |
|  |  | Domestic life | *3，6，13，23* | *3: “I was very down because I feel kind of too young to feel . . . to be useless in society and even to my family. I want to be with my young granddaughter that I love dearly and I don’t have the energy to babysit her as often as I’d like to and play with her on*  *the floor, and now with my access I can’t even lift her up.”*  *6: “You’ve lost your independence... you depend on other people all the time.”* |
|  |  | Social activities | 5，6，9，13，23 | *9: “Peter, dealing with depression after his sudden kidney failure, distanced himself creating a chasm of space between himself and others: ‘**I started getting cranky at home and... I didn’t want to talk to nobody. I didn’t want to associate with the family in any way. I just wanted to be left alone’.”*  *13: “In my personal family we are used to saying, oh let’s go to the movies or let’s go hang out. I know I get tired and we can’t take that long walk. So, I either get excluded or the entire trip gets derailed and I feel bad about that.”* |
| **Experiences of decision-making** | **Struggling to make a decision** | Hesitated and confused | 16，19 | *16: “He [Name of Nephrologist] brought up dialysis and was asking me whether I want to have peritoneal dialysis or haemodialysis. During that conversation we seemed to conflict with each other, so what I thought was one thing, he said, “No, no, no, that’s not what you want...”and I’m like, “No, I’m pretty sure I want that.”* |
|  |  | Delay in making decisions | 7，10，12，16 | *7: “I have just been informed about these things and then I think it has been part of a common understanding that it was going to be peritoneal dialysis. But I think we haven’t discussed it and said that we have not agreed upon anything, per se. So there is not that kind of clarity.”* |
|  |  | An urgent choice | 10，11 | *10: “Tests deteriorated to the point that it was needed, had it been put off about for 2-3 months. Felt tired and weak.”* |
|  | **Shared model** | Patient autonomy | 2，4，7，11，17 | *11: “‘Chose this one [PD], and probably there isn’t any difference, but in my heart, I felt as though that this was better. And so did my primary [care physician], when I talked to him too, and I’ve known him for years’ (65–70-year-old man, PD).”*  *17: “. . . the more information you have, it is important. Even though the doctors are helping you, if you don’t help yourself, you are not going to go anywhere.”* |
|  |  | Building mutual trust | 7，8，14，17 | *8: “For us, trust is very important. How they approach us is another factor, to reduce barriers, to form trust.”* |
|  |  | Iterative decision process | 19 | *19: “So we have had the number of meetings we think we needed. But we don’t know yet. The difficult thing here is that we are talking about something we imagine. It’s like a trip we’re going on. Then you have some expectations, but you don’t know where it really ends. It’s like when we start this, we’ll get to know something, and it’s great, what we are told, but we don’t know if there will be any questions along the way, and there automatically will be. Afterwards, we had the experience, we bought the trip or we have been on it…”* |
|  |  | Necessary information and guidance | 1，4，7，18，19 | *1: “They understood where I was coming from straight away wanting CAPD. They could see why I could choose it, that it did fit in with my life better and, if anything, I’d made up my mind anyway and after I was talking to them, it confirmed it.”* |
|  |  | Decision aid | 19 | *19: “But when you sit there starry-eyed and don’t know anything, it [the decision aid] can help a lot. Also, that you get more information about it [the decision].”* |
|  | **Paternalistic model** | Patient lacks decisional power | 8，11，21 | *11: “I did not want to [start dialysis]. I was quarrelling with my physician, because he was really... interested in having my dialysis. I didn’t like it... They didn’t give me very much information.”* |
|  |  | Inappropriate information | 8，11，22 | *8: “Sometimes it’s easy to overwhelm people with too much information, if you get too much information you just don’t know where to start.”* |
|  |  | Depending on professional expertise | 2，3，11，12 | *2: “I would hope that health care providers are sufficiently trained to inform the patients at the right time what to expect and not wait until the very last minute.”* |
|  | **Informed model** | Decision-making power in patients | 21 | *21:* *“They must be allowed to make informed choices. They must know why we do things and why they get this treatment, the point of coming here so frequently, the point of the medications.”* |
|  |  | Thorough information | 21 | *21: “Laypeople don’t usually know anything about kidney failure and dialysis, or the difference between HD and PD – it all depends on what information they get… and we think it’s okay to start with PD…so I would say it’s a slightly steered choice.”* |
|  |  | Physicians' influence | 11，21 | *21: “As a rule, we let patients choose the modality they want. Of course, you can try to influence them a bit towards the direction you think wise. If people are active, we promote home-based treatment. However, the patients themselves should make the final choice.”* |
|  | **Aspects of modality choice** | Illness experiences | 1，8，14，15 | *8: “I thought my family and my boy and what was best for us, my family doesn’t do well when I have to come into hospital, that was also a big consideration.”*  *14: “I knew some old people in town who had been on dialysis and they always looked terrible and died, I thought it was the dialysis that made them look terrible, and made them die, that’s what lots of people think.”* |
|  |  | Impacts on life | 1，3，8，12，15 | *12: “It is more so the inconvenience of it because I’m very active within the community and although I’m retired—nevertheless it’s—I suppose in a sense it’s an intrusion into the normal daily life. Especially if you’re going to be travelling around the place, around New Zealand or overseas, although we don’t do that all that much now, we used to do a far bit.”*  *15:* *“when he gets up in the morning, he’ll start his day as per normal so that’s not going to affect his daily routine.”* |
|  |  | Concerns regarding treatment | 1，8，15 | *1: “When she rolled the sleeves up on her blouse [to show fistula] I won’t say I couldn’t believe what I saw, that’s not true. It was almost like watching a horror film...I mean it’s unfortunate for anybody who’s around me, because I turned around straight away and says, ‘and you chose to have this?’.”* |
|  |  | Resources | 8，15 | *8: “We have such a tight budget now, to add anything extra even five dollars of power, that could tip us over, so that meant the machine at home was out.”* |
| **Engagement in self-care** | **Modifying health behaviours** | Adhering to diet and fluid recommendation | *5，13，18，21，23* | *13:* *“My problem is liquids, as long as I have been on dialysis that is the one thing that is the most difficult. I have tried everything. I have tried sucking on ice, buying a certain water bottle, a certain size cup.”*  *23: “I thought to myself that I could eat more if I didn’t increase my weight; I could eat less if my blood potassium was not controlled.”* |
|  |  | Adhering to medication regimen | 18，21 | *18: “We are all people, regardless of an illness or not. We become affected by various things in life. But participation means to be master of the illness instead of that the illness controls you. You have an illness you have to consider, but you can still control your life if you become more involved.”* |
|  | **Monitoring complications** | Measuring basic signs | 23 | *23: “Blood vessel care is very important. I am afraid of the occlusion of the AV shunt at night, and I don’t even dare to sleep. It would result in me being unable to ‘wash’ my kidneys (dialysis). I want to use an L-shaped pillow to prevent occlusion. When I wake up, I will touch the blood vessel. If the flow is not strong, I will strengthen my grasping muscles and let the blood flow improve.”* |
|  |  | Staying alert for symptoms | 5，6，18，23 | *6: “‘The dialysis drains you right out... I was worrying about my blood pressure... I can feel it, you get headaches...’. Sylvia experienced fatigue, ‘I go home and sit in my armchair and go to sleep.”* |
|  | **Keeping regular dialysis** | Taking part in planning care and treatment | 18 | *18: “One patient said: ‘Yes, it is a stronger sense of freedom when you can manage the time for the dialysis sessions. If you are on a trip for two days you can change your time for dialysis and then you are not so tied up.”* |
|  |  | Self-mastering dialysis operation | 18，20，21 | *18: A variety of actions can be performed, representing patient participation, such as trimming the dialysis machine with the necessary devices, and/or self‐cannulation. This is considered to be advanced patient participation by HCPs, requiring self‐confidence and knowledge, the latter acquired by means of attaining information, and engaging in learning opportunities.* |
| **Facilitators for patient engagement** | **Basic capability to engage** | Patient's physical function | 20 | *20: At the onset of kidney failure, accepting one’s illness and the necessity of dialysis may require one’s full attention; in the acute phase, the patient often has severe symptoms, such as overwhelming fatigue, limiting the patient’s opportunities to engage in activities.* |
|  |  | Cognitive function | 2，18，20，24 | *24: “I really do. I really do. I just feel like if I can follow what they’re telling me to do, well, then I can continue maybe a little longer.”* |
|  |  | Psychological function | 3，13，14，23 | *3: “You’ve got to learn to deal with your situation. I just started reading up on it and Dr X helped me out from there . . . more in education. You have to get your act together and start thinking about yourself and your family.”* |
|  | **Appropriate education** | More personalized information | 2，3，10，17，18 | *17: “Basically it boils down to that they don’t want to scare the patients . . . I have had doctors tell me one thing and because I have been through it, I knew they were trying to fluff it up so you wouldn’t feel bad . . . the patient’s not making an informed decision. They are only hearing one side of it.”* |
|  |  | Timely and common language | 2，3，4，20 | *20: “You can talk more about it the next time and let it take time because patients are different and can be affected in various ways.”* |
|  | **Supportive relationships** | Therapeutic alliance | 2，4，10，18，19，20 | *4: “She was very, very good because she came to my house and explained things first of all... I think it’s a good idea because it doesn’t come as such a shock then.”*  *18: “Meanwhile, you talk about so much more. You get to know each other and I think it makes it easier for patients to participate, when you know each other//and you become more open and tell how you feel.”* |
|  |  | Social support | 1，4，5，11，14，17，19 | *1: “It’s still a big decision to make. They are putting themselves at risk ‘cos my brother is only 22 and he’s fit and he’s well and him giving me his kidney is going to make him not well. And I think he’s barmy doing it.”* |
|  |  | Peer support | 1，4，5，7，8，14，19，20，22 | *1: “When I walked into CAPD it felt just right. I felt part of that talk. I though ‘yes, they are talking about me, this is what I want’. And I knew that. But with haemo, it was just like being there and listening to his experiences but not feeling part of it or wanting to be part of it.”*  *14: “They walk you through it. I learnt a lot in those sessions. Because it’s you’re your own culture, I guess. You just can see the reality there. I learnt a lot from those classes, more than talking to a doctor.”* |
|  | **Values and resources** | Healthcare team's consensus of "patient engagement" | 20，21，22 | *20: If an HCP believes that the engagement of patients is of limited value, the HCP will not engage in guiding the patient to an increased health literacy, which will constrain the patient’s opportunities to participate.* |
|  |  | Time for patient engagement | 17，20，21，22 | *21: If you are concerned with people and your patients, you should be concerned with patient participation as well. However, it takes more of your time. If you just decide on behalf of the patients, you get things done faster.* |
|  |  | Program for patient engagement | 10，17，20，21 | *20: The kidney program offers such ventures to patients in pre-dialysis, including classes where patients meet on a regular basis with the HCPs on the dialysis team and more experienced patients.* |
